# Complete genome sequence of phi29-like *Microbacterium foliorum* podovirus phage PineapplePizza

**DOI:** 10.1128/MRA.00478-23

**Published:** 2023-09-06

**Authors:** Kylie LaBianca, Jessica Butura, Arden McKnight, Karin Pan, Sean Whelan, Elizabeth Punska, Robert Pause, Bright Igbinosa, Alexander Ribbe, Peter Chien, Daniel Russell, Rebecca Garlena, Graham Hatfull, Deborah Jacobs-Sera, Jessica Rocheleau

**Affiliations:** 1 Department of Biochemistry and Molecular Biology, University of Massachusetts, Amherst, Massachusetts, USA; 2 Department of Biology, University of Massachusetts, Amherst, Massachusetts, USA; 3 Department of Polymer Science and Engineering, University of Massachusetts, Amherst, Massachusetts, USA; 4 Department of Biological Sciences, University of Pittsburgh, Pittsburgh, Pennsylvania, USA; Department of Biology, Queens College, Queens, New York, USA

**Keywords:** bacteriophages

## Abstract

Bacteriophage PineapplePizza is a podovirus infecting *Microbacterium foliorum* NRRL B-24224. The genome is 16,662 bp long and contains 23 predicted protein-coding genes. Interestingly, PineapplePizza shows amino acid similarities to well-studied *Bacillus subtilis* phage phi29.

## ANNOUNCEMENT

Bacteriophages infecting *Microbacterium* hosts are substantially diverse (1) and may be useful for bioremediation (2), *Microbacterium* genetics, and treatment of *Microbacterium* infections (1, 3). Of the sequenced phages infecting *Microbacterium foliorum*, four are singletons with no close relatives (4). Here we describe PineapplePizza, a singleton bacteriophage that infects *M. foliorum* and shares genomic features with *Bacillus subtilis* phage phi29.

PineapplePizza was isolated from soil in Amherst, Massachusetts, USA (global positioning system or GPS 42.3747N, 72.5196W) using standard methods (5).

**Fig 1:**
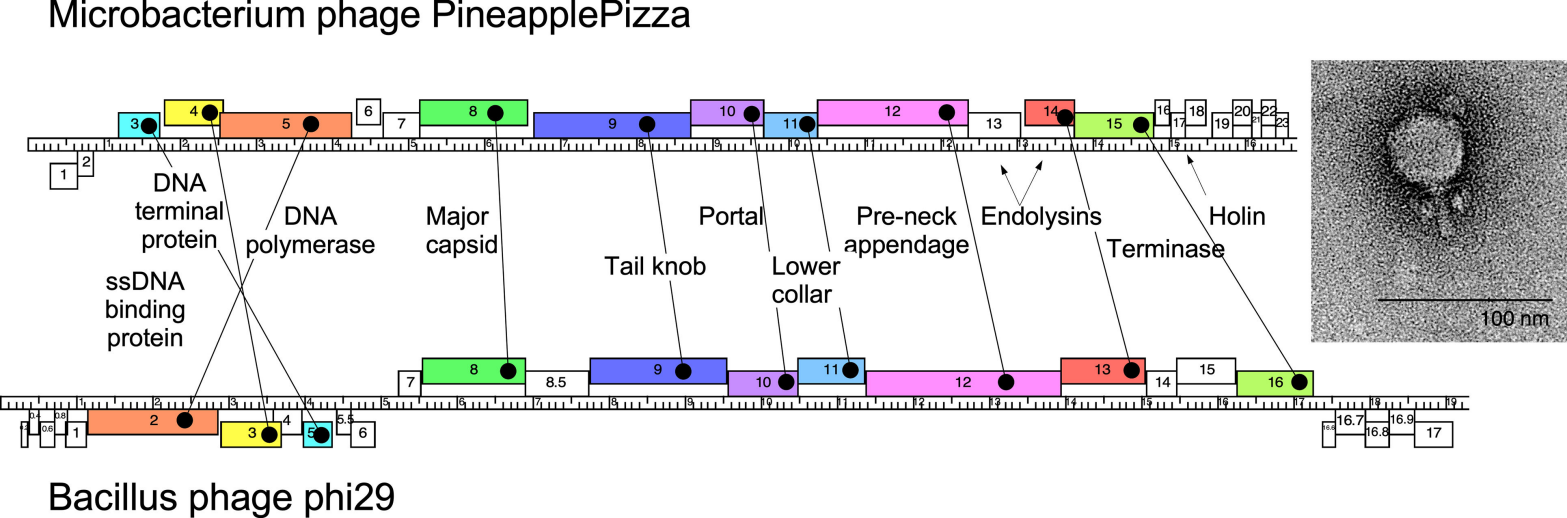
Genome organization of *Microbacterium* phage PineapplePizza as compared to *Bacillus* phage phi29 (EU771092). The phage genome, as depicted by the ruler in the center of the figure, is displayed with genes represented by boxes above and below, reflecting rightward and leftward transcription, respectively. Gene boxes with color represent homologs to phi29, as defined in Table 1. The homologs are connected with black lines. Inset is of the phage particle using negative-stain transmission electron microscopy.

Soil was suspended in peptone-yeast extract-calcium (PYCa) liquid medium, filtered through a 0.22-µm filter, and the filtrate plated in top agar with *Mi. foliorum* NRRL B-24224 and incubated at 30°C. PineapplePizza was purified with two rounds of plating and formed bullseye-type plaques with an average outer diameter of 2.3 mm after 48 h at 30°C. Negative-stain transmission electron microscopy showed PineapplePizza has podovirus morphology with capsids 40–43 nm wide and 42–48 nm long (Fig. 1, *n* = 6) and a tail length of 25 nm as measured using ImageJ v1.53r21 (6).

DNA was extracted from high titer lysates using a zinc chloride precipitation method (7), prepared for sequencing using the NEBNext Ultra II Kit (New England Biolabs, Ipswich, MA), and sequenced using an Illumina MiSeq instrument (v3 reagents) at the Pittsburgh Bacteriophage Institute (Pittsburgh, PA). Sequencing was performed to 9,214-fold coverage from 1,056,847 total single-end 150 bp reads. Assembly and quality control checks were performed with Newbler v2.9 and Consed v29.0, respectively (8, 9). The genome of PineapplePizza has 16,662 base pairs and a G + C content of 53.6%. No sequencing reads continued past the genome ends, and a 101-bp inverted repeat at the genome ends is consistent with covalently bound terminal proteins, as in phi29 (10). Whole-genome alignment with NCBI BLASTn (11) showed no significant nucleotide similarity to other *Microbacterium* phages, and PineapplePizza was classified as a singleton. The genome of PineapplePizza was autoannotated using Glimmer v3.02 (12) and GeneMark v2.5 (13), and then manually refined using Phamerator (14), DNA Master v5.23.6 (http://phagesdb.org/DNAMaster/), PECAAN, BLAST (11), and HHPred (15). No tRNA genes were identified by Aragorn v1.2.38 (16) or tRNAscan-SE v2.0 (17). All analyses were conducted using default settings.

PineapplePizza has 23 protein-encoding genes, of which 11 were assigned a function, and 15 were orphans (Table 1; Fig. 1). All but the first two genes are rightward-transcribed, consistent with architectural features of *Microbacterium* phages (Fig. 1) (1). PineapplePizza has 10 genes homologous to *Bacillus* phage phi29 as determined by HHPred (probability >97%, *E* value <6E−5, Table 1), including a ssDNA binding protein, DNA terminal protein, DNA polymerase, terminase, and six structural protein genes. A cryo-electron microscopy study of phi29 shows near-atomic detail of the entire bacteriophage particle (18) allowing for speculation that PineapplePizza has a similar structure. The tail knob, lower collar, and pre-neck appendage proteins found in phi29-family phages have not previously been identified in phages infecting *Microbacterium* hosts.

**TABLE 1:** *Microbacterium* PineapplePizza gene functions and homologs

Gene	Direction	Function	Phi29 homolog	Supporting data source hits^*a*^	*E* value	ID/SIM^*b*^
1	R	Hypothetical protein	–	NA	NA	NA
2	R	Hypothetical protein	–	NA	NA	NA
3	F	ssDNA binding protein	phi29_5	PF17427.3	1e−38	13/28
4	F	DNA terminal protein	phi29_3	PF05435.14	2e−34	19/37
5	F	DNA polymerase	phi29_2	2PY5_B	2e−55	37/53
6	F	Hypothetical protein	–	NA	NA	NA
7	F	Hypothetical protein	–	NA	NA	NA
8	F	Major capsid protein	phi29_8	6QZ0_7I	9E−75	24/45
9	F	Tail knob protein	phi29_9	5FB4_A	1E−101	20/36
10	F	Portal protein	phi29_10	PF05352.15	2E−61	25/43
11	F	Lower collar protein	phi29_11	6QZ9_0 J	2E−53	25/40
12	F	Pre-neck appendage protein	phi29_12	3GQ8_A	9E−14	17/29
13	F	Endolysin, protease M15 domain	–	NA	NA	NA
14	F	Endolysin, protease M23 domain and cell wall binding domain	phi29_13	P15132	6E−5	15/30
15	F	Terminase	phi29_16	P11014	6E−57	30/49
16	F	Hypothetical protein	–	NA	NA	NA
17	F	Holin	–	NA	NA	NA
18	F	Hypothetical protein	–	NA	NA	NA
19	F	Hypothetical protein	–	NA	NA	NA
20	F	Hypothetical protein	–	NA	NA	NA
21	F	Hypothetical protein	–	NA	NA	NA
22	F	Hypothetical protein	–	NA	NA	NA
23	F	Hypothetical protein	–	NA	NA	NA

^*a*^ Supporting Data Source hits found at HHPred (15) with Pfam database v35, PDB_mmCIF70_14_Apr, and UniProt-SwissProt_viral70_3_Nov_2021.

^*b*^ Needleman-Wunch global alignment percentage identity/similarity (NCBI: gap open/extend penalties = 11/1).

^*c*^ – indicates that no phi29 homolog was detected.

^*d*^ NA indicates not applicable.

## Data Availability

PineapplePizza is available at GenBank with Accession No. ON724010 and Sequence Read Archive (SRA) No. SRX14483237.

## References

[1] Jacobs-Sera D, Abad LA, Alvey RM, Anders KR, Aull HG, Bhalla SS, Blumer LS, Bollivar DW, Bonilla JA, Butela KA, Coomans RJ, Cresawn SG, D’Elia T, Diaz A, Divens AM, Edgington NP, Frederick GD, Gainey MD, Garlena RA, Grant KW, Gurney SMR, Hendrickson HL, Hughes LE, Kenna MA, Klyczek KK, Kotturi H, Mavrich TN, McKinney AL, Merkhofer EC, Moberg Parker J, Molloy SD, Monti DL, Pape-Zambito DA, Pollenz RS, Pope WH, Reyna NS, Rinehart CA, Russell DA, Shaffer CD, Sivanathan V, Stoner TH, Stukey J, Sunnen CN, Tolsma SS, Tsourkas PK, Wallen JR, Ware VC, Warner MH, Washington JM, Westover KM, Whitefleet-Smith JL, Wiersma-Koch HI, Williams DC, Zack KM, Hatfull GF. 2020. Genomic diversity of bacteriophages infecting Microbacterium spp. PLOS ONE 15:e0234636. doi:10.1371/journal.pone.0234636 32555720 PMC7302621

[2] Ali N, Dashti N, Khanafer M, Al-Awadhi H, Radwan S. 2020. Bioremediation of soils saturated with spilled crude oil. Sci Rep 10:1116. doi:10.1038/s41598-019-57224-x 31980664 PMC6981149

[3] Amano J, Hase R, Otsuka Y, Tsuchimochi T, Noguchi Y, Igarashi S. 2019. Catheter-related bloodstream infection by Microbacterium paraoxydans in a pediatric patient with B-cell precursor acute lymphocytic leukemia: a case report and review of literature on Microbacterium bacteremia. J Infect Chemother 25:806–810. doi:10.1016/j.jiac.2019.03.013 30982728

[4] Russell DA, Hatfull GF. 2017. PhagesDB: the actinobacteriophage database. Bioinformatics 33:784–786. doi:10.1093/bioinformatics/btw711 28365761 PMC5860397

[5] Poxleitner M, Pope WH, Jacobs-Sera D, Sivanathan V, Hatfull GF. SEA-PHAGES phage discovery guide. HHMI SEA-PHAGES Phage Discov Guide.

[6] Schneider CA, Rasband WS, Eliceiri KW. 2012. NIH image to Imagej: 25 years of image analysis. Nat Methods 9:671–675. doi:10.1038/nmeth.2089 22930834 PMC5554542

[7] Santos MA. 1991. An improved method for the small scale preparation of bacteriophage DNA based on phage precipitation by zinc chloride. Nucleic Acids Res 19:5442–5442. doi:10.1093/nar/19.19.5442 1656393 PMC328918

[8] Russell DA. 2018. Sequencing, assembling, and finishing complete bacteriophage genomes, p. 109–125. In Clokie, MRJ, Kropinski, AM, Lavigne, R (eds.), Bacteriophages. Springer New York, NY. 10.1007/978-1-4939-7343-9_9 29134591

[9] Gordon D, Green P. 2013. Consed: a graphical editor for next-generation sequencing. Bioinformatics 29:2936–2937. doi:10.1093/bioinformatics/btt515 23995391 PMC3810858

[10] Ito J. 1978. Bacteriophage phi29 terminal protein: its association with the 5′ termini of the phi29 genome. J Virol 28:895–904. doi:10.1128/JVI.28.3.895-904.1978 731797 PMC525814

[11] Altschul SF, Gish W, Miller W, Myers EW, Lipman DJ. 1990. Basic local alignment search tool. J Mol Biol 215:403–410. doi:10.1016/S0022-2836(05)80360-2 2231712

[12] Delcher AL, Bratke KA, Powers EC, Salzberg SL. 2007. Identifying bacterial genes and endosymbiont DNA with Glimmer. Bioinformatics 23:673–679. doi:10.1093/bioinformatics/btm009 17237039 PMC2387122

[13] Besemer J, Borodovsky M. 2005. GeneMark: web software for gene finding in prokaryotes, eukaryotes and viruses. Nucleic Acids Res 33:W451–4. doi:10.1093/nar/gki487 15980510 PMC1160247

[14] Cresawn SG, Bogel M, Day N, Jacobs-Sera D, Hendrix RW, Hatfull GF. 2011. Phamerator: a bioinformatic tool for comparative bacteriophage genomics. BMC Bioinformatics 12:395. doi:10.1186/1471-2105-12-395 21991981 PMC3233612

[15] Söding J, Biegert A, Lupas AN. 2005. The HHpred interactive server for protein homology detection and structure prediction. Nucleic Acids Res 33:W244–8. doi:10.1093/nar/gki408 15980461 PMC1160169

[16] Laslett D, Canback B. 2004. ARAGORN, a program to detect tRNA genes and tmRNA genes in nucleotide sequences. Nucleic Acids Res 32:11–16. doi:10.1093/nar/gkh152 14704338 PMC373265

[17] Lowe TM, Eddy SR. 1997. tRNAscan-SE: a program for improved detection of transfer RNA genes in genomic sequence. Nucleic Acids Res 25:955–964. doi:10.1093/nar/25.5.955 9023104 PMC146525

[18] Xu J, Wang D, Gui M, Xiang Y. 2019. Structural assembly of the tailed bacteriophage Φ29. Nat Commun 10:2366. doi:10.1038/s41467-019-10272-3 31147544 PMC6542822

